# Paracetamol vs. Ibuprofen in Preterm Infants With Hemodynamically Significant Patent Ductus Arteriosus: A Non-inferiority Randomized Clinical Trial Protocol

**DOI:** 10.3389/fped.2020.00372

**Published:** 2020-07-17

**Authors:** Ana García-Robles, Ana Gimeno Navarro, María del Mar Serrano Martín, María José Párraga Quiles, Anna Parra Llorca, José Luis Poveda-Andrés, Máximo Vento Torres, Marta Aguar Carrascosa

**Affiliations:** ^1^Neonatal Research Group, Health Research Institute La Fe, University and Polytechnic Hospital La Fe, Valencia, Spain; ^2^Division of Neonatology, University and Polytechnic Hospital La Fe, Valencia, Spain; ^3^Division of Pharmacy, University and Polytechnic Hospital La Fe, Valencia, Spain; ^4^Division of Neonatology, Regional University Hospital of Malaga, Málaga, Spain; ^5^Division of Neonatology, University Hospital Reina Sofía, Córdoba, Spain

**Keywords:** ductus, paracetamol, efficacy, safety, pharmacokinetics, pharmacogenetics

## Abstract

**Background:** Currently, the first line treatment of persistent ductus arteriosus (PDA) is either indomethacin or ibuprofen. However, the potentially life-threatening side effects associated to their use have prompted physicians to look for alternative options. The incorporation of paracetamol as an alternative to ibuprofen in the management of PDA is still based on insufficient clinical evidence. Hence, more clinical trials are needed to establish a therapeutic role for paracetamol in the management of PDA that take into consideration short- and long-term safety and efficacy outcomes.

**Study Design:** This is a non-inferiority, randomized, multicenter, double-blinded study to evaluate the efficacy, and safety of intravenous (IV) paracetamol vs. IV ibuprofen (standard treatment) for PDA in preterm patients with a gestational age ≤ 30 weeks. At baseline, patients will be randomized (1:1) to treatment with paracetamol or ibuprofen. The primary endpoint is closure of the ductus after the first treatment course. Secondary endpoints are related to effectiveness (need for a second treatment course, rescue treatment, reopening rate, time to definitive closure, need for surgical ligation), safety (early and long-term complications), pharmacokinetics, and pharmacodynamics, pharmacogenetics, pharmacoeconomics, and genotoxicity. Long-term follow-up to 24 months of corrected postnatal age will be performed using Bayley III neurodevelopmental scale.

**Trial Registration:** ClinicalTrials.gov Identifier: NCT04037514. EudraCT: 2015-003177-14.

## Background

Ductus arteriosus (DA) is an essential vascular shunt during fetal life that connects the pulmonary artery with the aorta (1). Under physiologic conditions, the DA closes spontaneously a few hours after birth, leading to the complete independence of the systemic and pulmonary circulations. Incidence of failure of the DA to close after birth is inversely proportional to gestational age (GA), with an incidence ranging from 10 to 20% in preterm neonates >32 weeks to 60% in those <28 weeks of gestation (2–4). When the ductus remains open, a portion of the circulating blood volume is redirected from the systemic to the pulmonary circulation. Depending on the size of the ductus, the diverted flow may cause pulmonary overflow and impaired end-organ perfusion. This hemodynamic situation is known as hemodynamically significant patent ductus arteriosus (hsPDA). HsPDA is associated with an increased risk of potentially severe clinical complications such as necrotizing enterocolitis (NEC), intraventricular hemorrhage (IVH), bronchopulmonary dysplasia (BPD), periventricular leukomalacia (PVL), acute renal failure, and death (5, 6). Therefore, recognizing and effectively treating hsPDA is a key point in the management of premature infants.

The first line therapy for hsDPA is a non-steroidal anti-inflammatory drug (NSAID), either indomethacin or ibuprofen. For patients who don't respond to NSAIDs or for whom pharmacologic treatment is contraindicated, surgical ligation is performed (7–9). The adverse effects of surgical management, although not frequent, are potentially severe. They may include reversible complications such as pneumothorax, infection, hemorrhage, or chylothorax, and/or irreversible complications such as vocal cord or diaphragmatic paralysis (1).

Indomethacin, a potent prostaglandin inhibitor, has traditionally been the drug of choice in the treatment of hsPDA. However, despite its established efficacy, its use has been linked to complications related to decreased cerebral, renal, and mesenteric perfusion (1). Ibuprofen has shown similar efficacy rates of up to 80% and lower hemodynamic effects compared to indomethacin. However, renal and mainly gastrointestinal complications are still present, such as NEC or intestinal perforation (10, 11).

Paracetamol, or acetaminophen, has recently emerged as an alternative to ibuprofen. This approach was first published in 2011, when Hammerman et al. (12) reported a case series of use of paracetamol as treatment of hsPDA in five neonates who had either failed or had contraindications to ibuprofen therapy. Rate of ductus closure was 100%, with no adverse events reported. In subsequent years, additional case series and clinical trials evaluating this new treatment option have been published (7, 12–30).

Table 1 summarizes the existing randomized clinical trials (RCTs) comparing paracetamol to standard treatment. Based on the data provided by these studies, paracetamol appears to have promising clinical results with a low rate of side effects.

**Table 1:** Randomized clinical trial: paracetamol vs. active drug (ibuprofen or indomethacin).

**References**	**Drug**	**Administration**	**Dosage**	***N***	**% total closure rate**	**Serum level**	**Hepatotoxicity**
Dang et al. (13)	Paracetamol	Oral	15 mg/kg/6 h	80	81.2	No	No
	Ibuprofen	Oral	10–5–5 mg/kg	80	78.8	No	No
Oncel et al. (14)	Paracetamol	Oral	15 mg/kg/6 h	40	96.6	No	No
	Ibuprofen	Oral	10–5–5 mg/kg	40	93.6	No	No
El-Mashad et al. (30)	Paracetamol	IV	15 mg/kg/6 h	100	88	No	No
	Ibuprofen	IV	10–5–5 mg/kg	100	83	No	No
	Indomethacin	IV	10–5–5 mg/kg	100	87	No	No
Bagheri et al. (7)	Paracetamol	Oral	15 mg/kg/6 h	67	91	No	No
	Ibuprofen	oral	20–10–10 mg/kg	62	90.3	No	No
Yang et al. (27)	Paracetamol	Oral	15 mg/kg/6 h	44	70.5	No	No
	Ibuprofen	Oral	10–5–5 mg/kg	43	76.7	No	No
Dash et al. (28)	Paracetamol	Oral	15 mg/kg/6 h	38	100	No	No
	Indomethacin	IV	0.2 mg/kg/ 24 h	39	94.6	No	No
Al-lawama et al. (29)	Paracetamol	Oral	10 mg/kg/6 h	13	92	No	No
	Ibuprofen	Oral	10 mg/kg/24 h	9	89	No	No
Kumar et al. (31)	Paracetamol	Oral	15 mg/kg/6 h	81	78	No	No
	Ibuprofen	Oral	10–5–5 mg/kg	80	81	No	No

The systematic review published in 2016 by Terrin et al. included two RCTs and 14 uncontrolled studies (32). It found no difference in the rate of ductal closure when paracetamol was used in place of ibuprofen (risk ratio [RR] 1.07, 95% CI 0.87–1.33 after 3 days of treatment, RR 1.03, 95% CI 0.92–1.16 after 6 days of treatment). In addition, safety profiles of paracetamol and ibuprofen were similar. The results are limited, however, by the poor quality of the included studies.

In 2018, Huang et al. (33), published a systematic review of five RCTs including a total of 677 neonates treated with either paracetamol or ibuprofen. The rates of primary and overall PDA closure were similar between treatments (RR 1.03, *p* = 0.56 and RR 1.02, *p* = 0.62 for paracetamol and ibuprofen, respectively). No differences were observed in the incidence of PDA complications: NEC (RR 0.86, *p* = 0.70), IVH (RR 0.84, *p* = 0.55), BPD (RR 0.69, *p* = 0.16), ROP (RR 0.58, *p* = 0.15), sepsis (RR 0.88, *p* = 0.48), or death (RR 1.45, *p* = 0.45). However, paracetamol showed a trend toward a reduced risk of renal failure (RR 0.20, *p* = 0.07), and a significantly reduced risk of gastrointestinal bleeding (RR 0.28, *p* = 0.009).

In 2018, Jasani et al. (34) performed a meta-analysis including RCTs comparing paracetamol to any cyclooxygenase (COX) inhibitor. Six RCTs were identified, involving 688 neonates treated with either paracetamol or ibuprofen. No differences in PDA closure were observed after the first course of treatment [RR 0.90, 95% confidence interval (CI) 0.71–1.13]. However, neonates treated with paracetamol had a lower incidence of gastrointestinal hemorrhage (RR 0.28; 0.12–0.69), acute renal impairment or increased serum bilirubin. No significant differences in alanine aminotransferase (ALT) or clinical outcomes such as NEC, BPD, IVH, ROP, pulmonary hemorrhage, surgical ligation, or mortality were assessed. The same meta-analysis examined two RCTs comparing paracetamol to indomethacin among 273 enrolled neonates. No differences in PDA closure were observed after the first course of treatment (RR 0.96; 0.55–1.65). In a pooled analysis of seven RCTs comparing paracetamol to any COX inhibitor no differences in PDA closure rate were observed among 861 neonates after the first treatment course (RR 0.90; 0.72–1.13), and paracetamol treatment was associated with a lower rate of gastrointestinal hemorrhage (RR 0.51; 0.28–0.91). No differences were observed in rates of NEC, ROP, BDP, IVH, pulmonary hemorrhage, surgical ligation, or mortality (34).

The Cochrane Systematic Review performed in 2018 by Ohlsson et al. (35) included eight studies that reported data collected on 916 infants. Studies that achieved at least moderate-quality evidence according to the GRADE classification suggested that paracetamol is as effective as ibuprofen; the group of low-quality evidence studies suggested that paracetamol is more effective than placebo or no intervention and also that paracetamol is as effective as indomethacin in PDA closure. In view of these results, Ohlsson et al. (35) concluded that paracetamol appears to be a promising alternative to indomethacin or ibuprofen for PDA closure, potentially with fewer adverse effects. However, further research regarding the effectiveness and safety of paracetamol is needed before the evidence is definitively established or rejected.

Moreover, there are no data published regarding neurodevelopment follow-up in patients receiving paracetamol.

In summary, published systematic reviews and meta-analyses conclude that the existing evidence is still not sufficient to establish a therapeutic role for paracetamol in the treatment of hsPDA and additional larger trials are required, with special focus on developmental consequences associated with the use of this drug.

In addition to the lack of definitive clinical evidence supporting its use in hsPDA, there is also insufficient knowledge about the pharmacokinetics and pharmacodynamics of paracetamol in the neonatal period, especially in patients with hsPDA.

The aim of this study is to demonstrate the non-inferiority of paracetamol compared with ibuprofen and to address the safety and cost-effectiveness of this treatment in premature infants.

## Methods

### Study Design and Population

#### Study Design

This is a randomized, multicenter, double-blinded non-inferiority study to evaluate the efficacy, and safety of IV paracetamol (intervention) vs. IV ibuprofen (standard treatment) for the treatment of hsPDA in preterm neonates. Patients will be randomized (1:1 ratio) to the paracetamol or ibuprofen group. The study will be conducted at four hospitals: University and Polytechnic Hospital La Fe (Valencia, Spain), Regional University Hospital of Malaga (Málaga, Spain), University Hospital Reina Sofía (Córdoba, Spain), and Cabueñes University Hospital (Gijón, Spain).

#### Study Population

Preterm infants with GA ≤ 30 weeks with diagnosis of hsPDA based on clinical suspicion and confirmed by echocardiogram performed by a pediatric Cardiologist will be eligible for the study.

The definition of “Hemodynamically significant PDA” has been selected from the most common and reliable echocardiographic parameters widely used to consider the treatment of the PDA (36). It is defined as a ductal diameter >1.5–2.0 mm and at least one of the following:

◦ Continuous flow through DA.
◦ Retrograde diastolic flow in the descending aorta.
◦ Dilation of the left atrium, defined as left atrial/aortic ratio (LA/AO) > 1.5 mm (measured on M-mode echocardiogram)
◦ Ductus size/descending aorta diameter ratio > 0.5 mm.

Inclusion and exclusion criteria are described in Table 2 and comprise mainly the contraindications of ibuprofen in this population.

**Table 2:** Inclusion and exclusion criteria of the study.

**Inclusion criteria**	**Exclusion criteria**
Written informed consent of parents/guardians	Major congenital malformations or chromosomopathies
GA ≤ 30 weeks	Imminent death
Postnatal age ≤ 2 weeks	Impossible or erroneous randomization
Need for ventilatory support	Participation in another clinical trial with medication
Birth or arrival in participating hospital within the period of application of the treatment	Diuresis <1 mL/kg/h in the 8 h prior to treatment or creatinine >1.8 mg/dL
First episode of hsPDA	Platelets <50,000/μL or active hemorrhage (tracheal, digestive, or renal)
	Recent (past 48 h) IVH (grades 3–4)
	Septic shock
	Severe hyperbilirubinemia or severe coagulopathy or liver failure
	Active NEC or intestinal perforation

Subjects will be screened to determine whether they meet all the inclusion criteria and have none of the exclusion criteria.

The study will only include hsPDA requiring treatment in the first 2 weeks of life, as the odds of closure decrease with time (4).

#### Objectives

The primary objective of the study is to evaluate the efficacy of IV paracetamol vs. standard IV ibuprofen treatment for PDA closure.

Secondary objectives are: (i) to compare the safety of both treatments; (ii) to improve the knowledge of pharmacokinetics, pharmacodynamics, and pharmacogenetics of paracetamol and ibuprofen in the neonatal period; (iii) to make a pharmacoeconomic evaluation of the use of both drugs; and finally (iv) to perform a genotoxicity study of administered drugs.

#### Primary Outcome

The primary outcome is the rate of hsPDA closure after one round of treatment with paracetamol (experimental drug) vs. ibuprofen (control drug). A ductus will be considered to be closed when the diameter measures <1 mm on echocardiography performed by a pediatric cardiologist.

#### Secondary Outcomes

##### Outcomes related to effectiveness

- Need for a second treatment course
- Closure rate after two treatment courses
- Need for rescue treatment after two courses of treatment
- Rate of ductus reopening after closure
- Closure rate after reopening
- Time to ductus closure
- Need for surgical ligation.

##### Outcomes related to safety

- Incidence of early complications (occurring during the course of treatment): renal failure, NEC, IVH, hyperbilirubinemia, bleeding, gastrointestinal perforation
- Incidence of late complications (over the course of the admission): BPD, PVL, NEC, neonatal retinopathy, sepsis, death.

##### Outcomes related to pharmacokinetics and pharmacodynamics

- Determination of serum levels of paracetamol achieved with standard doses
- Pharmacodynamic model of paracetamol in the context of hsPDA
- Relationship of effectiveness/adverse reactions to serum levels
- Quantification of metabolites in urine and their relationship with drug elimination.

##### Outcomes related to pharmacogenetics

- Determination of genetic polymorphisms in TFAP2B, TGFBR2, EPAS1, MD-2, and GM2A genes in dry blood spots (DBS) and their relationship to efficacy or incidence of adverse reactions.

##### Outcomes related to pharmaco-economics

- Price-effectiveness ratio, assessed via a cost-effectiveness analysis accounting for observed efficacy.

##### Outcome related to genotoxicity

- Percent DNA damage.

#### Sample Size Calculation and Power

For the estimation of the sample size, data from previous studies (13, 14) were used to establish a Gaussian with mean 0.15 and standard deviation 0.3 for the coefficient that determines the log-odds of closure of the paracetamol group with respect to the ibuprofen group. Assuming this previous distribution of the log-odds, that the ibuprofen group produces closure in 80% of the cases, and establishing the inferiority limit for the paracetamol group in 70% of closures (−10%), it has been estimated that 150 patients per group would be needed to establish the non-inferiority of the paracetamol treatment compared to the ibuprofen treatment with a statistical power of 80 and 95% credibility.

La Fe and Carlos Haya University Hospitals are a reference centers in their areas having 6,000–6,500 births per year and patients referred from other centers. They have ~100–150 preterm admissions <1,500 gr per year. In addition, there is other two recruiting centers (Reina Sofía Hospital and Cabueñes University Hospital) included in the study, so we estimate that the sample size could be accomplished in the 3 years period of the study duration.

The study is also designed to perform a pharmacoeconomic analysis of the treatment. In the case that paracetamol arises as non-inferior to ibuprofen, that is, they are assumed to have the same therapeutic effectiveness, we will perform a cost-minimization analysis, calculating, and comparing the costs associated with each therapeutic strategy based in the cost per unit (laboratory sale price), preparation cost and administration cost. If both drugs are not equivalent in efficacy, we will perform a cost-effectiveness analysis using the closure rate after the first round of treatment and the rate of occurrence of clinically relevant adverse reactions related to treatment and score in the Bayley III test. The incremental cost and the incremental cost-effectiveness ratio will be calculated.

### Treatment of Subjects

#### Intervention

The paracetamol group will receive IV doses of 15 mg/kg IV every 6 h for 3 days. The ibuprofen group will receive an initial dose of 10 mg/kg IV followed by 5 mg/kg IV at 24 and 48 h (three doses are considered a treatment course). Given that the treatments have different dosing schedules, to maintain blinding, patients in the ibuprofen group will receive an equivalent volume of placebo (glucose 5%, normal saline or according to the center's usual practice) at 6-h intervals to correspond with the dosing times of paracetamol. If the duct remains open >1 mm after the full treatment course has been completed, another course of the same drug will be administered (maximum two courses).

**Figure 1:**
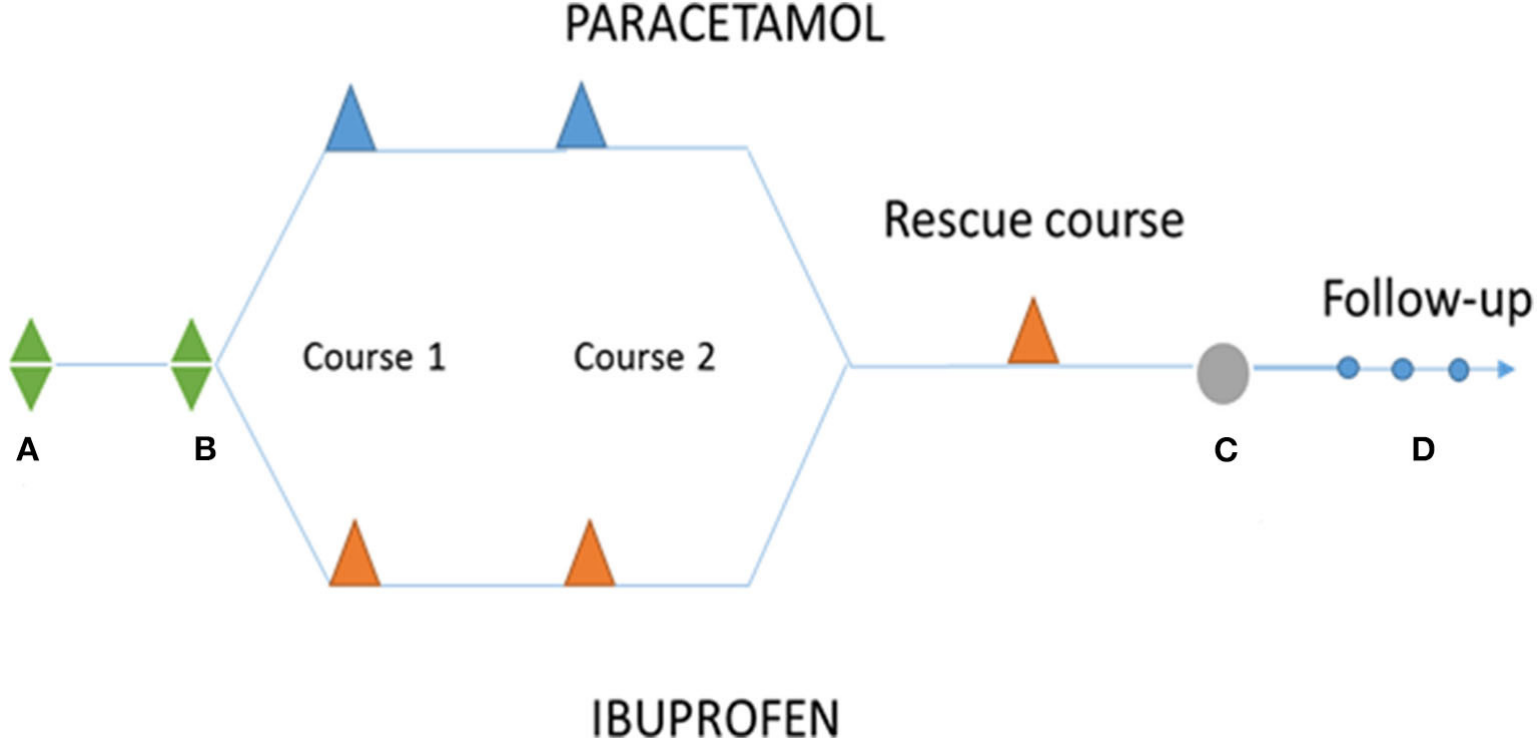
Diagram of study. **(A)** Birth; **(B)** diagnostic and randomization; The PARACETAMOL group will receive IV doses of 15 mg/kg administered every 6 h for 3 days (maximum two courses = 6 days of treatment). The IBUPROFEN group (control group) will receive an initial dose of IV 10 mg/kg followed by 5 mg/kg at 24 and 48 h (the three doses are considered a treatment course, maximum two courses). **(C)** Surgical close. **(D)** FOLLOW-UP: 40 weeks, 12 months, and 24 months.

If the medical treatment fails (defined as the ductus measuring >1 mm after completion of two rounds of treatment), a course of ibuprofen (not blinded) at usual doses will be considered in both groups with the intention of offering at least one course of the standard treatment to all patients before considering surgery. If this rescue course fails, surgical closure will be pursued if deemed appropriate. Figure 1 described diagram of study.

The paracetamol dose of 15 mg/kg every 6 h was chosen based on previously-reported data for paracetamol in the treatment of hsPDA in neonates (12–14, 16, 17, 26, 37).

#### Assessments During Study Treatment

##### Related to efficacy

If possible, daily echocardiography will be performed in order to monitor ductus diameter and timing of closure. Compulsory echocardiography will be performed at diagnosis and at the end of each treatment course.

##### Safety assurance

Daily blood tests including creatinine, platelet count, bilirubin, and liver enzymes will be performed in conjunction with routine samples to rule outside effects due to the medication.

##### Pharmacokinetic study

In hemodynamically stable patients, blood samples will be obtained to determine the serum levels of drugs during treatment to develop a population pharmacokinetic model for IV paracetamol in premature infants with hsPDA. On the 1st day, samples will be taken at 30 min and at 1, 3, 6, and 12 h. In the subsequent days, samples will be taken at 24, 48, and 72 h following the first dose. The blood volume per sample will be 100–200 μL. On the 1st day of the study, sampling will be limited to patients with a central catheter, from whom blood can be obtained without venipuncture.

Non-linear mixed-effects models will be constructed from paracetamol serum concentration–time data in NONMEM v7.3. Covariates will include the number of doses administered, hour of sample extraction, body weight, gestational age, postnatal age, sex, creatinine, total bilirubin, and estimated glomerular filtration rate.

Urine will be collected once the first course of treatment begins and within 24 h of completion of the last dose to measure drug metabolites.

##### Pharmacogenetics analysis

A blood spot will be deposited in a WhatmanTM 903 card or similar once during the study and left to dry at room temperature. The collector cards will be stored at room temperature until analysis. The genomic DNA will be extracted by the method validated by Ramos et al. (38), based on an alkaline lysis to obtain the genetic information, then subjected to PCR amplification with primers responsible for amplifying the polymorphism to be determined. The genetic polymorphisms in the TFAP2B, TGFBR2, EPAS1, MD-2, and GM2A genes will be determined. The collector card and the leftover pellets of the plasmatic samples of the pharmacokinetic analysis will be saved for further determinations of other polymorphisms that may be useful, such as AGTR1, TRAF1, etc. or others that may add to our knowledge of relevant pathology.

##### Genotoxicity of drugs

The genomic damage caused by the treatments with paracetamol and ibuprofen will be determined using a modification of the alkaline electrophoresis of individual cells, or “comet assay,” with repair enzymes for the detection of specific lesions in the DNA in the pellet (polymorphonuclear cells). The repair enzyme that we will use in our study is formamido-pyrimidine-DNA glycosylase (FPG), which recognizes oxidized purines. The comet assay is a well-established approach for detecting genotoxicity from drugs.

#### Follow-Up Evaluations

The study will incorporate follow-up visits at 40 weeks, 12 months, and 24 months of corrected age. At each visit, weight, height, and head circumference will be measured and a complete structured physical evaluation of the child will be performed. In the third follow-up visit, a comprehensive neurodevelopmental assessment using Bayley III-ES, GMFCS, and sensorial visual and auditory acuity will be performed.

### Procedures

#### Recruitment and Consent

Once a potential study participant has been identified, investigators will explain the nature of the study to the infant's parents or legal guardians and answer any questions they may have regarding the study. Written informed consent will be obtained before the start of any study-related procedures and will be reviewed, signed and dated by investigator and parents/legal guardians. The information sheet will include all the complementary tests that will be carried out during the study. A separate consent will be signed to obtain pharmacogenetics samples.

#### Randomization, Blinding, Treatment Allocation, and Administration

Randomization will be carried out by the biostatistician of Health Research Institute La Fe using the R^®^ 3.5.1 software (R Development Core Team, Auckland, New Zealand). The biostatistician will provide the randomization list to the Pharmacy Department. Patient numbers will be assigned sequentially in order of entry into the study. Patients will be randomly assigned to either the paracetamol or ibuprofen group using randomization by blocks and stratified by GA (24^+0^–26^+6^ and 27^+0^–30^+6^).

The drug solutions will be prepared in a daily basis including weekends by the Pharmacy Department in indistinguishable syringes to keep the study blind. Four numbered syringes with the daily treatment for each patient will be prepared every day. Administration will proceed sequentially, beginning with syringe 1. In the ibuprofen group, syringe 1 will contain ibuprofen, while syringes 2–4 contain a placebo.

Due to the incompatibility of ibuprofen with other medications and parenteral nutrition, no other medications or parenteral nutrition should be administered within 15 min of syringe 1. There are no similar compatibility concerns with either paracetamol or the placebo, so syringes 2–4 can be administered either alone or via Y-site as a short infusion over 15 min, preferably undiluted.

##### Withdrawal of subjects

Parents/legal guardians can withdraw consent at any time and for any reason if they wish to do so without any consequences.

The researcher may also withdraw a patient from the study at any time if he believes study procedures have not been followed, for the benefit of the patient, or in the event of unacceptable levels of toxicity.

The reasons for withdrawal will be registered in the electronic Case Report Form (eCRF). All infants who leave the study will receive treatment as per standard unit practice.

#### Unblinding

The blinding can be opened in emergency cases in which the knowledge of the assigned treatment is essential for the medical care and well-being of the patient. In this case, the date and reason for the unmasking must be noted in the eCRF by the researcher.

The researcher or neonatologist in charge will communicate with the Pharmacy Department regarding treatment provided to the patient.

Once the final patient recruited into the study has been discharged from the hospital, the blinding will be lifted to allow for statistical analysis of the results. The neurodevelopmental specialist who will provide long-term follow-up will remain blinded until all follow-up assessments have been completed.

#### Adverse Events (AEs)

An AE is any harmful and unintended reaction to a drug under investigation.

AE will be monitored until 28 days after the end of the treatment period and/or until all AE-related consultations for the patients have been resolved. Data of all study participants will be included in the safety analyses. We will not consider those conditions commonly related with prematurity, such as jaundice, apneic-bradycardic syndrome, anemia, or electrolyte, and glucose abnormalities, to be AEs.

A serious adverse event (SAE) is any untoward medical occurrence that results in death, is life-threatening, requires inpatient hospitalization, or prolongation of existing hospitalization, results in persistent or significant disability/incapacity, or is a congenital anomaly/birth defect. In the event of SAE occurs, the researcher will inform the promoter (La Fe Research Institute) within a maximum period of 24 h from the moment the event is identified, completing, and signing the SAE notification form.

During the study, the promoter will prepare yearly safety reports following the recommendations indicated in the International Conference on Harmonization (ICH) E2F guide and will present them to the regulatory authorities and to the relevant Institutional Review Board (IRB) following the schedule established in the current legislation.

#### Statistical Analysis

The analytic strategy has been based on the intention-to-treat principle. Categorical variables are described as the numerical count (percentage) of each category, and are compared with Pearson's chi-squared test or Fisher's exact test. Continuous variables are represented by a box-and-whisker plot. If the continuous variables are normally distributed (*p* > 0.05 in the Shapiro-Wilk test), they are described as the mean ± standard deviation, and are compared using Student's *t*-test, first testing the hypothesis of equality of variances using the test of Levene. If they are not normally distributed, they are described as the median (p25, p75) and compared using the Mann-Whitney *U*-test. Comparison of repeated measures between the two groups is performed using two-way analysis of variance (ANOVA), first testing the Mauchly sphericity hypothesis. Survival times are expressed as the median time (95% CI), and the comparison between groups is performed with the log-rank test. Throughout the study, *p* < 0.05 is accepted as the limit of statistical significance. The magnitude of the effect is quantified with the risk difference (expressed as a percentage), and its accuracy is indicated with a 95% CI.

#### Data Handling and Study Monitoring

A record in an eCRF must be completed for each patient included in the study. Data will be anonymized using a unique code for each participant. The key document that will contain the name of each patient related to the code number will be stored in the folder of the principal investigator (PI) at each center. Data will be kept in an institutional research location of the PI, secured with a password or key for the period specified by legislation.

Periodic monitoring visits will be carried out during the trial by an external monitor independent from the research team to ensure that the protocol and good clinical practices are being followed. The monitors will be able to review the source documents to confirm that the data collected in the eCRFs are accurate. The researcher and the institution guarantee the monitors direct access to the source documents and to the relevant regulatory authorities for verification.

#### Ethical Considerations

All the procedures have been reviewed and approved by the IRB of the PI's hospital (Comité de Ética e Investigación Médica (CEIm); University and Polytechnic Hospital, Valencia, Spain) and the Approval Number is: 5/27-06-2018/439) also by the local IRBs of the participating hospitals. It has been also approved by the Spanish Drug Agency as per legal requirement.

According to good-practice guidelines, blood samples for pharmacokinetics will be only obtained if a central catheter in place is available and the total amount of blood per day will be limited to 1 mL/kg/day.

The study will be conducted in accordance with the protocol, ICH guidelines, the applicable regulations, and guidelines governing the conducting of clinical studies in Spain, and the ethical principles originating from the Declaration of Helsinki.

## Discussion

Many Neonatal intensive care units (NICUs) consider off-label use of paracetamol in hsPDA cases where ibuprofen (the current first line option) is contraindicated or has proven inefficient. This therapeutic approach has yielded promising results, with high rates of ductus closure and a good safety profile. However, in our own experience, efficacy rates of paracetamol have been much lower than those reported (non-published data). This discrepancy may be related to many factors, including the low publication index of studies with negative results compared with studies with positive results [publication bias (39)], or data from studies that report on several individuals with unclear selection approach [possible selection bias (40)].

We choose a non-inferiority study because is the best way to demonstrate that the experimental treatment is not unacceptably less effective than an existing treatment. With this design, we are able to collect data regarding potential advantages over the established treatment, such as a lower incidence of adverse reactions or a more favorable cost-effectiveness profile. It is known that the efficacy of ibuprofen in PDA closure is 70–85% (41), but it is associated with significant renal and mainly gastrointestinal, such as NEC and intestinal perforation (10, 11). Paracetamol safety is well-documented, as it is a common drug used for treating fever and pain in infants and children. If non-inferiority is demonstrated, the lower incidence of side-effects and the lower cost would make paracetamol an ideal drug for neonates with hsPDA.

Of published clinical trials, only study of Kumar et al. (31, 42) has a non-inferiority design but administration of treatment was oral and patients included were <32 weeks of gestation. One of the main inconveniences of this design is the large population size needed to demonstrate non-inferiority. To date, our sample size is the biggest of reported trials (35).

Failure of DA closure after birth is inversely proportional to GA, with incidence ranging from 10 to 20% in preterm neonates >32 weeks to 60% in those <28 weeks of gestation (2–4). We chose GA ≤ 30 weeks because it includes the majority of newborns requiring treatment for this condition, thus increasing the external validity and allowing our results to be generalized to this population at large. A more narrow GA range, as chosen in other trials, limits external generalization. Using randomization by blocks and stratifying by GA limits bias and further enhances the applicability of our results.

Pharmacologic management of PDA is not without risk, and limiting treatment to only those neonates with hsPDA is the most appropriate strategy for balancing the benefits of treatment with the risks of potential adverse effects (43–45). Therefore, the diagnosis of hsPDA should rely on objective parameters. There is no consensus for what constitutes a hemodynamically significant PDA and therefore when is most appropriate to treat a PDA in preterm infants. We have chosen published criteria with high sensitivity and specificity to define when a PDA should be classified as hemodynamically significant (46, 47). These include a ductal diameter >1.5–2.0 mm and at least one of the following: continuous flow through the DA, retrograde diastolic flow in the descending aorta, LA/AO ratio > 1.5, or ductus size/descending aorta diameter ratio >0.5.

The primary outcome of our study is the rate of closure of the hsPDA after a single course of treatment. For the purpose of our study, closure is defined as a ductus diameter <1 mm on echocardiography, as defects off this size are typically not hemodynamically significant and in most cases proceed to complete closure. In some published studies (28), it is not clear how many treatment courses were required for ductal closure, or if the rate of the ductus reopening differs between treatments, so potential bias can be found in the results. The appropriate duration of treatment of PDA with paracetamol has not yet been established, and in our study echocardiogram will be performed every day of treatment in order to help to resolve this question. We believe that this information will improve future PDA management and will give us useful information to avoid unnecessary or excessive treatment.

Paracetamol, a non-classical NSAID, is an analgesic and antipyretic agent that has weak antiplatelet and anti-inflammatory activities. It reduces the synthesis of prostaglandins by inhibiting prostaglandin synthetase (PGHS), an enzyme in the peroxidase (POX) region instead of the COX region, as is the case of ibuprofen or indomethacin (12).

Paracetamol is metabolized almost exclusively by the liver (90–95%) and eliminated mainly as paracetamol-glucuronide (47–62%), paracetamol-sulfate (47–62%), and, to a lesser extent, cysteine conjugates (48).

Age-related changes in bioavailability, metabolism, and the rate of elimination of paracetamol take place during childhood. These changes are particularly evident during infancy. Neonates have lower metabolic and elimination capacities than older infants, and varying rates among different subjects is explained by covariates, such as size or weight, as well as different disease characteristics. Preterm neonates have a higher distribution volume, lower elimination rate, and higher half-life values for plasma concentration of paracetamol than older infants (48). Neonates, infants and children up to 10 years old eliminate a significantly lower amount of glucuronide conjugates and more sulfate conjugates than adults (49).

At present, there is scarce information available regarding the paracetamol plasma concentration required for PDA closure. Plasma levels of paracetamol for analgesic and antipyretic effects range from 10 to 30 μg/mL (15, 37, 48, 50). However, only three studies have addressed paracetamol plasma concentrations in the management of hsPDA (15, 37, 50). The small number of patients enrolled in these studies doesn't allow for conclusions regarding the efficacy of paracetamol related to its plasma concentration. Recently, Bin-Nun et al. (51) reported the association between serum paracetamol concentration measured at steady-state (they chose 4 h after the 8th dose) and ductal closure in 10 neonates treated with oral paracetamol (15 mg/kg/q6h). A paracetamol concentration >20 mg/L had 100% sensitivity and specificity for ductal closure. The El-Khuffash's study (16) showed that the clinical efficacy of paracetamol in PDA closure may depend on the duration of treatment, the dose and the mode of administration. This would suggest that a critical serum concentration of paracetamol is needed in order to achieve a maximum therapeutic effect.

Because there is no established critical serum concentration of paracetamol, our study is designed to confirm a therapeutic threshold of serum concentration required for ductal closure, identify optimal timing for evaluation of serum concentration and relate concentration to gestational age, treatment duration, and paracetamol metabolism (assessed via urine metabolites) to optimize treatment success. Moreover, although paracetamol dosage was chosen according to previous reported studies (12–14, 16, 17, 26, 37), it was adopted without appropriate pharmacokinetic or pharmacodynamic studies to establish safety and efficacy, and our study will add this information regarding the paracetamol dosage most used for treatment of hsPDA.

Regarding the route of administration, the protocol followed in our NICU is administered via IV, since the oral route is not always an option in neonates. The data in favor of orally- vs. IV- administered paracetamol have not yet been fully confirmed. We believe that route of administration may be very relevant, as the IV route is likely more suitable than in this population due to the frequency of feeding intolerance and intestinal complications and in whom enteral absorption is uncertain. Moreover, the oral preparation is hyperosmolar thus should be used with caution when infants are NPO or allowed only low-volume intakes (52). Some authors suggest that the slower rate of absorption of oral paracetamol relative to IV paracetamol could lead to a longer exposure of the ductus to the drug and a greater response rate (53). Singla et al. (54) administered a single dose of intravenous, oral, or rectal paracetamol to adults, and intravenous paracetamol achieved faster and higher plasma concentrations.

One of the greatest concerns when administering paracetamol to neonates is the possible hepatotoxicity due to the toxic metabolite N-acetyl-p-benzoquinone-imine (NAPQI) (53). In general, toxicity is lower in neonates. They have relatively low levels of CYP2E1 enzymatic activity so their oxidation of paracetamol is slower, forming smaller amounts of toxic metabolites. In addition, neonates have higher rates of glutathione synthesis (53). However, extremely preterm infants (<28 weeks) have a limited capacity for glutathione synthesis due to the lack of expression of the enzyme gamma-cystathionase in the trans-sulfuration pathway and therefore limited or no ability to synthesize L-cysteine, a component of the tripeptide glutathione (55). According to published literature, no signs of hepatotoxicity have been reported during PDA treatment (34).

In conclusion, between the available drugs for PDA treatment, paracetamol seems to be a promising alternative to NSAIDs. Most authors agree that there is a need of better designed trials to establish its efficacy, short- and long-term safety and neurodevelopmental outcomes.

Our study is an adequately-powered RCT that will allow for the establishment of paracetamol as standard therapy for the management of PDA and to definitively establish its safety and efficacy. The ultimate aim would be to achieve an individualized therapeutic approach, selecting the best treatment according to the patient's characteristics and including pharmacologic aspects aiming to reduce toxicity.

## Ethics Statement

The studies involving human participants were reviewed and approved by University and Polytechnic Hospital, Valencia, Spain. Written informed consent to participate in this study was provided by the participants' legal guardian/next of kin.

## Author Contributions

AG-R, AG, MS, MP, AP, JP-A, MV, and MA were all involved in development of the study protocol. AG-R prepared the initial draft of the manuscript. AG-R, AG, and MA set up the database infrastructure for the intervention. All authors read, contributed to editing, and approved the final manuscript.

## Conflict of Interest

The authors declare that the research was conducted in the absence of any commercial or financial relationships that could be construed as a potential conflict of interest.
