# Corrigendum: Paracetamol vs. Ibuprofen in Preterm Infants With Hemodynamically Significant Patent Ductus Arteriosus: A Non-inferiority Randomized Clinical Trial Protocol

**DOI:** 10.3389/fped.2021.834454

**Published:** 2022-01-13

**Authors:** Ana García-Robles, Ana Gimeno Navarro, María del Mar Serrano Martín, María José Párraga Quiles, Anna Parra Llorca, José Luis Poveda-Andrés, Máximo Vento Torres, Marta Aguar Carrascosa

**Affiliations:** ^1^Neonatal Research Group, Health Research Institute La Fe, University and Polytechnic Hospital La Fe, Valencia, Spain; ^2^Division of Neonatology, University and Polytechnic Hospital La Fe, Valencia, Spain; ^3^Division of Pharmacy, University and Polytechnic Hospital La Fe, Valencia, Spain; ^4^Division of Neonatology, Regional University Hospital of Malaga, Málaga, Spain; ^5^Division of Neonatology, University Hospital Reina Sofía, Córdoba, Spain

**Keywords:** ductus, paracetamol, efficacy, safety, pharmacokinetics, pharmacogenetics

In the original article, we neglected to include “Co-funded by European Regional Development Fund/European Social Fund “A way to make Europe”/“Investing in your future”” in the **Funding Statement**.

The authors apologize for this error and state that this does not change the scientific conclusions of the article in any way. The original article has been updated.

## Publisher's Note

All claims expressed in this article are solely those of the authors and do not necessarily represent those of their affiliated organizations, or those of the publisher, the editors and the reviewers. Any product that may be evaluated in this article, or claim that may be made by its manufacturer, is not guaranteed or endorsed by the publisher.

